# Prevalence of hypertension in Africa in the last two decades: systematic review and meta-analysis

**DOI:** 10.1093/cvr/cvaf125

**Published:** 2025-07-15

**Authors:** Paul Olowoyo, Akinkunmi Paul Okekunle, Osahon Jeffery Asowata, Segun Atolani, Moustafa I Morsy, Elisabetta Caiazzo, Bamba Gaye, David Danladi Kadan, Dario Bruzzese, Tomasz J Guzik, Pasquale Maffia, Mayowa O Owolabi

**Affiliations:** Department of Medicine, Federal Teaching Hospital, Ido-Ekiti, Nigeria; College of Medicine and Health Sciences, Afe Babalola University, Ado-Ekiti, Nigeria; Department of Food and Nutrition, Seoul National University, Seoul 08826, Republic of Korea; Translational Gerontology Branch, National Institute on Aging, Baltimore, MD, USA; Department of Epidemiology and Medical Statistics, University of Ibadan, Ibadan 200284, Nigeria; Department of Medicine, Federal Teaching Hospital, Ido-Ekiti, Nigeria; College of Medicine and Health Sciences, Afe Babalola University, Ado-Ekiti, Nigeria; School of Infection & Immunity, College of Medical, Veterinary and Life Sciences, University of Glasgow, G12 8TA Glasgow, UK; School of Infection & Immunity, College of Medical, Veterinary and Life Sciences, University of Glasgow, G12 8TA Glasgow, UK; Department of Pharmacy, School of Medicine and Surgery, University of Naples Federico II, 80131 Naples, Italy; Alliance for Medical Research in Africa (AMedRA), Dakar, Senegal; Institute of Health and Development (ISED), Cheikh Anta Diop University, Dakar, Senegal; Department of Biomedical Informatics, Emory University School of Medicine, Atlanta, GA, USA; Department of Epidemiology and Medical Statistics, University of Ibadan, Ibadan 200284, Nigeria; Department of Public Health, School of Medicine and Surgery, University of Naples Federico II, Naples, Italy; Centre for Cardiovascular Sciences, Queens Medical Research Institute, University of Edinburgh, Edinburgh, UK; Department of Internal Medicine, Centre for Medical Genomics Omicron, Jagiellonian University Collegium Medicum, Krakow, Poland; Africa-Europe CoRE in Non-Communicable Diseases & Multimorbidity, African Research Universities Alliance (ARUA), The Guild of European Research-Intensive Universities, Glasgow, UK; School of Infection & Immunity, College of Medical, Veterinary and Life Sciences, University of Glasgow, G12 8TA Glasgow, UK; Department of Pharmacy, School of Medicine and Surgery, University of Naples Federico II, 80131 Naples, Italy; Africa-Europe CoRE in Non-Communicable Diseases & Multimorbidity, African Research Universities Alliance (ARUA), The Guild of European Research-Intensive Universities, Glasgow, UK; School of Cardiovascular & Metabolic Health, College of Medical, Veterinary and Life Sciences, University of Glasgow, G12 8TA Glasgow, UK; Africa-Europe CoRE in Non-Communicable Diseases & Multimorbidity, African Research Universities Alliance (ARUA), The Guild of European Research-Intensive Universities, Glasgow, UK; Department of Medicine, College of Medicine, University College Hospital, University of Ibadan, Ibadan 200284, Nigeria; Center for Genomic and Precision Medicine, College of Medicine, University of Ibadan, Ibadan 200284, Nigeria; Blossom Specialist Medical Center, Ibadan 200285, Nigeria

**Keywords:** Hypertension, Cardiovascular diseases, Blood pressure, Africa

## Abstract

Despite being the most common cardiovascular risk factor, the actual burden of hypertension is poorly characterized in Africa. We meta-analysed the most extensive pooled data to determine the overall prevalence of hypertension in Africa. Following Preferred Reporting Items for Systematic Review and Meta-Analyses guidelines, we systematically searched Google Scholar, PubMed, ScienceDirect, and Web of Science databases to retrieve prevalence studies only on hypertension among Africans published between 2002 and 2023. Furthermore, we meta-analysed the crude and age-adjusted prevalences of hypertension using a random effect model due to the expected high heterogeneity, with logit transformation of the original proportions. Seventy-eight (out of an initial 779 screened) articles with complete data were included, with a total number of hypertension cases of 71 004 and a denominator population of 286 575, mostly from community-based studies in 23 countries. The pooled crude prevalence of hypertension was 28.5/100 persons [95% confidence interval (CI): 25.3–31.8%] and a 95% prediction interval of 7.6–65.6%; the pooled prevalence increased with age and was highest among the aged ≥75 years: 51.4% (95% CI: 42.0–60.6%) and remained highest in the Southern Africa region overall (34.8%) and in the last decade (2013–23; 44.5%). The point estimate of the pooled crude prevalence was higher among urban dwellers, 32.9% (95% CI: 26.8–39.5%), than rural residents, 26.3% (95% CI: 20.4–33.3%). In a subset of 21 articles reporting age stratification consistent with the World Health Organization standard population, the pooled age-standardized prevalence was 27.2/100 persons (95% CI: 20.9–33.6%). The burden of hypertension remains high, especially in urban areas and with increasing age. Frequent screening and treatment are recommended, especially in urban areas.

## Graphical Abstract

**Graphical Abstract:**
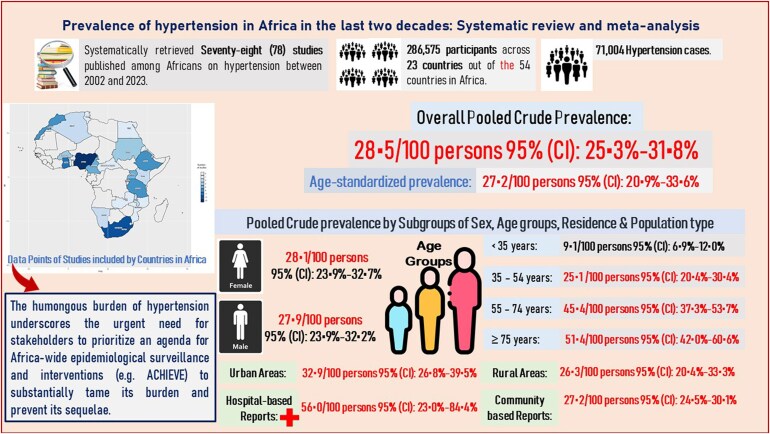
Graphical abstract of prevalence of hypertension in Africa in the last two decades.

## 1. Introduction

Hypertension is the most common modifiable risk factor for cardiovascular diseases (CVDs), with increasing prevalence and attributable mortality over the past decades in low- and middle-income countries (LMIC).^1,2^ The resulting CVD includes myocardial infarction, stroke, kidney disease, and peripheral arterial disease.^3^ The prevalence of these diseases, complicating hypertension, is also increasing.^4,5^ According to the Global Burden of Diseases in 2019, non-communicable diseases (NCDs) have outpaced communicable diseases as causes of death in Africa.^6^ Rapid urbanization and the adoption of unhealthy lifestyles are the major factors for the development of hypertension and other NCDs,^7^ which account for 15 million premature deaths each year among the elderly in high-income countries and the working class in LMIC, leading to high healthcare costs, limited ability to work, and financial insecurity. Furthermore, regional variations in cultural and social values with urbanization, economic growth, and development may affect the burden of hypertension in Africa.^8^ Despite being the most common cardiovascular risk factor, the actual burden of hypertension is poorly characterized in Africa.

Prior attempts to characterize the hypertension burden in this population were not sufficiently extensive and lacked the most pertinent and current evidence required for developing effective health interventions and programmes. In addressing these gaps, an extensive literature review is necessary for relevant estimates to capture the evolving landscape of hypertension differentials across the life course and by age groups, sex, and region of Africa. A detailed appraisal of the present epidemiological studies on hypertension is important for describing the regional prevalence and demographic pattern of hypertension to efficiently allocate the available limited resources for its control in Africa. Furthermore, this effort can provide evidence-based information to promote precision medicine approaches to achieve the collective regional and global health ambitions of improving African population health outcomes. Therefore, we conducted a systematic review and meta-analysis to characterize the prevalence of hypertension in Africa over the past two decades, stratified by age, sex, urban vs. rural settings, and regional variations.

## 2. Methods

This systematic review was reported using the Preferred Reporting Items for Systematic Review and Meta-Analyses (PRISMA) framework,^9^ and the meta-analysis was carried out in keeping with the Meta-analysis of Observational Studies in Epidemiology Guidelines.^10^ The protocol for this systematic review was prospectively registered in PROSPERO (Registration ID: CRD42021292327).

### 2.1 Search strategy and selection criteria

Four scientific databases, Google Scholar, PubMed, ScienceDirect, and Web of Science, were searched for previously published reports on the epidemiology of hypertension among Indigenous Africans from January 2002 to December 2022, and updated searches were conducted in 2023 using the search terms listed in Supplementary material online, *Table S1*.

### 2.2 Inclusion and exclusion criteria

Studies were eligible for inclusion if the population sample was strictly indigenous Africans aged (≥15 years) within geographical boundaries defined as Africa from community-based or hospital-based recruitment or registry with a clear definition of hypertension using standard criteria such as the International Society of Hypertension,^11^ American Heart Association,^12,13^ European Society of Cardiology,^14^ or World Health Organization (WHO).^15^ Studies were excluded if they were self-reported, case studies, randomized controlled trials, reviews, abstracts, or epidemiological reports from mixed populations without specific results for an African population or a clear description of the population and statistical estimates of interest. In addition, due to the dearth of longitudinal studies on the subject, cross-sectional studies were considered for inclusion only. All references were collated, and duplicates were removed manually.

### 2.3 Data collection, extraction, selection, and analysis

**Figure 1:**
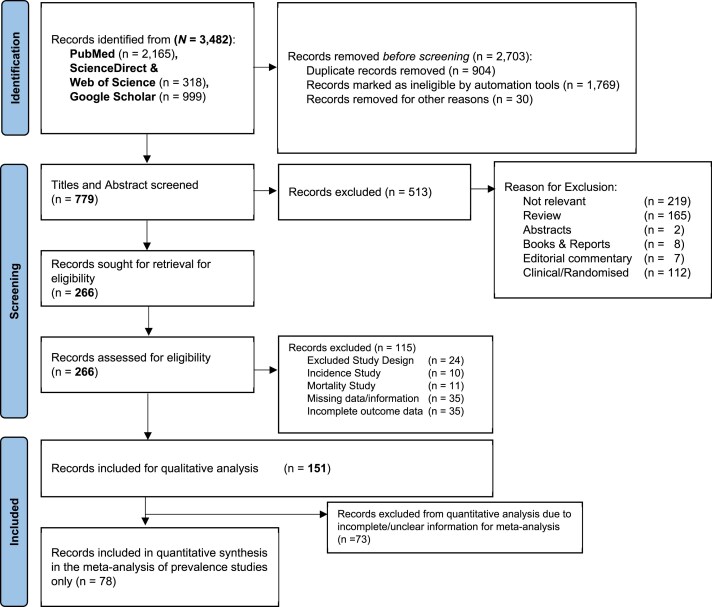
PRISMA flowchart of study selection.

Reviewers (P.O., A.P.O., O.J.A., and S.A.) independently screened the title or abstract of all citations, and A.P.O. evaluated the selected citations to resolve all disagreements. The article with the most comprehensive dataset was included, where there were multiple articles with overlapping datasets from the same sampling population. Details of the search strategy and criteria for article selection and inclusion have been summarized using the PRISMA flowchart in *Figure 1*. Also, O.J.A., D.D.K., and S.A. extracted data, which was carried out independently using a predefined proforma. M.I.M. and E.C. reviewed the extraction details, and the differences were resolved in an organized discussion in the review team (A.P.O., P.O., and O.J.A.) and authenticated by M.I.M. and E.C. Data extracted included the first author, year of publication, the study population characteristics (period, country, location, setting, age, and criteria for hypertension definition), and epidemiological data (number of hypertension cases by sex, residence, and age groups).

### 2.4 Statistical analysis

The prevalence of hypertension was the primary outcome. Data on the number of hypertension events and overall sample size were extracted from cross-sectional studies only to perform meta-analyses of the overall pooled prevalence for hypertension (with differences by sex: male vs. female; residence: rural vs. urban; age groups: 15–34; 35–54; 55–74, and 75+ years; and region of African: North, West, Central, East, and Southern Africa) fitting a random intercept logistic regression model with logit transformation of the single-study prevalence proportions. Results are reported as crude prevalence ratio (per 100 persons) with 95% confidence intervals (CIs). When possible, age-standardized prevalence using the WHO standard population^16^ was computed using a direct standardization method and meta-analysed. Between-study variance, i.e. tau squared, was estimated using restricted maximum likelihood. Due to the expected high heterogeneity among studies, 95% prediction intervals were computed, showing the expected range where 95% of future estimates would lie. Publication bias was evaluated using funnel plots (statistically assessed using Egger's regression intercept test). All statistical analyses were carried out using the ‘meta’ and ‘metafor’ packages in R software v 4.1.3,^17^ and a two-sided *P* < 0.05 was considered significant.

## 3. Results

### 3.1 Study characteristics and epidemiological data from included reports

**Figure 2:**
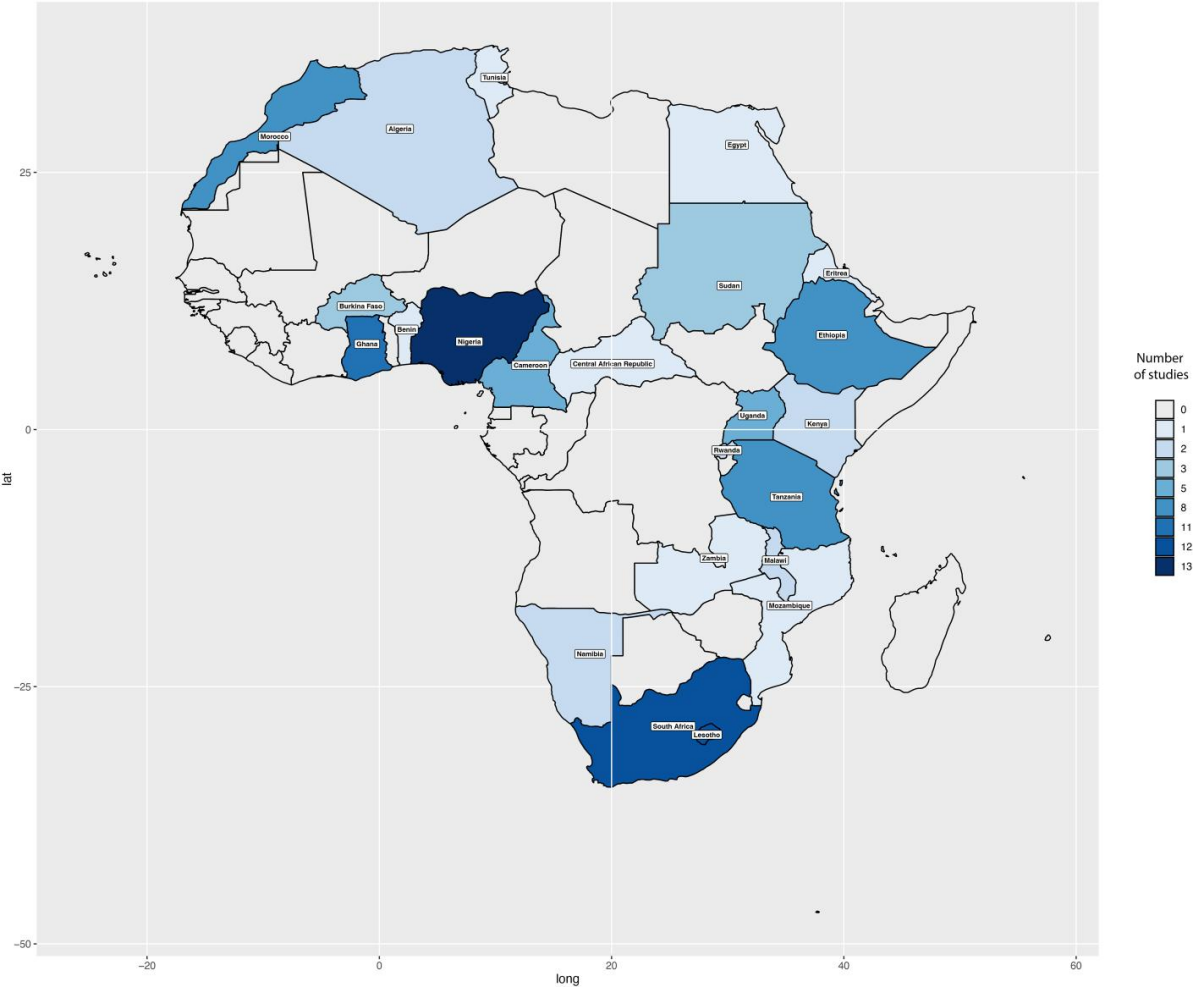
Geographic distribution of included studies assessing the crude prevalence of hypertension by countries of participant recruitment in Africa.

Of the 3482 records retrieved (*Figure 1*), 78 reports from 23 African countries (*Table 1*) were included. The earliest records were from Tanzania^48^ and South Africa,^49^ published in 2003; the most recent was published in 2023.^19^ Around half of the studies were conducted in both rural and urban areas, with approximately one-fourth of the studies primarily from urban areas only.^18,19,23,24,26,32,33,39,51,55,57,62,63,73,75,77,85–87,90,92^ Also, only five^19,27,43,55,59^ of the 78 reports were primarily hospital based (i.e. recruitment of participants was in a clinical setting or data from a hospital registry), and more than three-quarters (*n* = 72)^11,18,20–24,26,28,30–46,48–64,66–94^ of all studies defined hypertension primarily using blood pressure (BP) measurement in keeping with standard definitions. The total number of recorded hypertension cases during the reporting period was 71 004, with a population denominator (total sampled population) of 286 575.^11,18–94^ The geographical distribution of studies included by countries is in *Figure 2*.

**Table 1:** Summary of the characteristics of included studies on hypertension epidemiology in Africa

Study and publication year	Study period	Country	Location	Age group (years)	Location type	Study setting	Criteria for definition
Essayagh *et al*. 2021^18^	2021	Morocco	Oujda	≥18	Urban	Community-based	Systolic BP ≥ 140 mmHg and/or diastolic BP ≥ 80 mmHg
Boakye *et al*. 2023^19^	2021	Ghana^a^	Accra	≥18	Urban	Hospital-based	Self-report of hypertension diagnosis
Dai *et al*. 2022^20^	2007–10	Ghana	National survey	≥50	Multi-centre	Community-based	Systolic BP ≥ 130 and/or diastolic BP ≥ 80 mm/Hg
Koye *et al*. 2022^21^	2015	Ethiopia	National survey	15–69	Multi-centre	Community-based	Systolic BP ≥ 140 or diastolic BP ≥ 90 mmHg or treatment
Ntaganda *et al*. 2022^22^	2018	Rwanda	Kirehe	≥18	Rural	Community-based	Systolic BP ≥ 140 mmHg of diastolic BP ≥ 90 mmHg
Omar *et al*. 2022^23^	2019	Sudan	Gadarif	≥18	Urban	Community-based	Systolic BP ≥ 140 mmHg or diastolic BP ≥ 90 mmHg
Ayalew *et al*. 2022^24^	2021	Ethiopia	Wolaita Sodo	≥18	Urban	Community-based	Systolic BP ≥ 130 mmHg and/or diastolic BP ≥ 80 mmHg
Ntenda *et al*. 2022^25^	2016	South Africa	National survey	≥20	Both	Community-based	Self-report of hypertension diagnosis
Akinbule *et al*. 2022^26^	2022	Nigeria	Abeokuta	18–49	Urban	Community-based	Systolic BP ≥ 130 mmHg and/or diastolic BP ≥ 80 mmHg
Alami *et al*. 2022^27^	2017–18	Morocco^a^	Casablanca-Settat	≥20	Both	Hospital-based	Self-report of hypertension diagnosis
Fares *et al*. 2022^28^	2014–15	Egypt	National survey	18–59	Rural	Community-based	Systolic BP ≥ 130 mmHg and/or diastolic BP ≥ 80 mmHg
Dev *et al*. 2022^29^	2006–14	Multi-country	Multi-country survey	18–69	Both	Community-based	Self-report of hypertension diagnosis
Kuhudzai *et al*. 2022^30^	2017–18	South Africa	National survey	≥18	Both	Community-based	Systolic BP ≥ 140 mmHg only
Dorgbetor *et al*. 2022^31^	2021	Ghana	Multi-centre	15–49	Both	Community-based	Systolic BP ≥ 140 mmHg and/or diastolic BP ≥ 90 mmHg only
Tannor *et al*. 2022^32^	2019	Ghana	Kumasi	≥18	Urban	Community-based	Systolic BP ≥ 140 mmHg and/or diastolic BP ≥ 90 mmHg
Essa *et al*. 2022^33^	2020	Ethiopia	Debre Markos	≥18	Urban	Community-based	Systolic BP ≥ 140 mmHg and/or diastolic BP ≥ 90 mmHg andor antihypertensive medications
Plessis *et al*. 2022^34^	2010	South Africa	Multi-centre	≥35	Both	Community-based	Systolic BP ≥ 140 mmHg and/or diastolic BP ≥ 90 mmHg or antihypertensive medications
PauloseI *et al*. 2022^35^	2017	Ethiopia	Hawassa	≥31	Both	Community-based	Systolic BP ≥ 140 mmHg and/or diastolic BP ≥ 90 mmHg or antihypertensive medications
Teshome *et al*. 2022^36^	2020	Ethiopia	Multi-centre	≥18	Rural	Community-based	Systolic BP ≥ 140 mmHg and/or diastolic BP ≥ 90 mmHg andor antihypertensive medications
GelassaI *et al*. 2022^37^	2020	Ethiopia	Dano	19–65	Rural	Community-based	Systolic BP ≥ 140 mmHg and/or diastolic BP ≥ 90 mmHg
Moussouni *et al*. 2022^38^	2016–17	Algeria	National survey	18–69	Both	Community-based	Systolic BP ≥ 140 mmHg and/or diastolic BP ≥ 90 mmHg andor antihypertensive medications
Mouzouni *et al.* 2020^39^	2017–18	Morocco	Multi-centre	≥18	Urban	Community-based	Systolic BP ≥ 140 mmHg and/or diastolic BP ≥ 90 mmHg andor antihypertensive medications
Ellahi *et al*. 2022^40^	2018	Ghana	Hohoe	≥18	Both	Community-based	Systolic BP ≥ 140 mmHg and/or diastolic BP ≥ 90 mmHg only
Khamis *et al*. 2020^41^	2019–20	Tanzania	Monduli	≥18	Rural	Community-based	Systolic BP ≥ 140 mmHg and/or diastolic BP ≥ 90 mmHg andor antihypertensive medications
Ware *et al*. 2019^42^	2018	Ghana, South Africa	National Survey	≥18	Both	Community-based	Systolic BP ≥ 140 mmHg and/or diastolic BP ≥ 90 mmHg andor antihypertensive medications
Masilela *et al*. 2022^43^	2019	South Africa^a^	Eastern Cape Province	≥18	Both	Hospital-based	Systolic BP ≥ 140 mmHg and/or diastolic BP ≥ 90 mmHg only
Pengpid *et al*. 2022^44^	2017	Morocco	National Survey	≥18	NR	Community-based	Systolic BP ≥ 140 mmHg and/or diastolic BP ≥ 90 mmHg andor antihypertensive medications
Pengpid *et al*. 2022^45^	2017	CAR	Multi-centre	25–64	Both	Community-based	Systolic BP ≥ 140 mmHg and/or diastolic BP ≥ 90 mmHg only
Houehanou *et al.* 2022^46^	2018	Benin	Multi-centre	≥18	Both	Community-based	Systolic BP ≥ 140 mmHg and/or diastolic BP ≥ 90 mmHg andor antihypertensive medications
Brady *et al*. 2022^47^	2017	South Africa	National Survey	18–110	NR	Community-based	Self-report of hypertension diagnosis
Njelekela *et al*. 2003^48^	2000	Tanzania	Multi-centre	>30	Both	Community-based	Systolic BP ≥ 140 mmHg and/or diastolic BP ≥ 90 mmHg andor antihypertensive medications
Schutte *et al*. 2003^49^	1996–98	South Africa	North West Province	41–70	Both	Community-based	Systolic BP ≥ 140 mmHg and/or diastolic BP ≥ 90 mmHg only
Doulougou *et al*. 2014^50^	2013	Burkina Faso	Kaya	≥18	Both	Community-based	Systolic BP ≥ 140 mmHg and/or diastolic BP ≥ 90 mmHg andor antihypertensive medications
Mbouemboue *et al*. 2019^51^	2016	Cameroon	Ngaoundere	20–87	Urban	Community-based	Systolic BP ≥ 140 mmHg and/or diastolic BP ≥ 90 mmHg andor antihypertensive medications
Cappuccio *et al*. 2004^52^	2003	Ghana	Multi-centre	40–75	Both	Community-based	Systolic BP ≥ 140 mmHg and/or diastolic BP ≥ 90 mmHg andor antihypertensive medications
Muhihi *et al*. 2020^53^	NR	Tanzania	Morogoro	25–64	Rural	Community-based	Systolic BP ≥ 140 mmHg and/or diastolic BP ≥ 90 mmHg andor antihypertensive medications
Agyemang 2006^54^	2004	Ghana	Ashanti region	NR	Both	Community-based	Systolic BP ≥ 140 mmHg and/or diastolic BP ≥ 90 mmHg andor antihypertensive medications
Rguibi *et al*. 2007^55^	2001–02	Morocco^a^	Laayoune	≥20	Urban	Hospital-based	Systolic BP ≥ 140 mmHg and/or diastolic BP ≥ 90 mmHg only
Tesfaye *et al*. 2007^56^	2003–04	Ethiopia	Multi-centre	25–64	Both	Community-based	Systolic BP ≥ 140 mmHg and/or diastolic BP ≥ 90 mmHg andor antihypertensive medications
Temmara *et al*. 2007^57^	NR	Algeria	In-Salah	40–99	Urban	Community-based	Systolic BP ≥ 140 mmHg and/or diastolic BP ≥ 90 mmHg andor antihypertensive medications
Adedoyin *et al*. 2008^58^	NR	Nigeria	Ile-Ife	≥20	Both	Community-based	Systolic BP ≥ 140 mmHg and/or diastolic BP ≥ 90 mmHg only
Khalil *et al*. 2009^59^	1999–2001	Morocco^a^	Rabat	NR	NR	Hospital-based	Systolic BP ≥ 140 mmHg and/or diastolic BP ≥ 90 mmHg only
Tazi *et al*. 2009^60^	2000	Morocco	Multi-centre	≥20	Both	Community-based	Systolic BP ≥ 140 mmHg and/or diastolic BP ≥ 90 mmHg andor antihypertensive medications
Kengne *et al*. 2009^61^	1998	Cameroon	Bafut	≥15	Rural	Community-based	Systolic BP ≥ 140 mmHg and/or diastolic BP ≥ 90 mmHg and/or physician-diagnosed hypertension treated or not treated.
Njelekela *et al*. 2009^62^	NR	Tanzania	Temeke—Dar es Salaam	44–66	Urban	Community-based	Systolic BP ≥ 130 mmHg and/or diastolic BP ≥ 85 mmHg
Tesfaye *et al*. 2009^63^	NR	Ethiopia	Addis Ababa	25–64	Urban	Community-based	Systolic BP ≥ 140 mmHg and/or diastolic BP ≥ 90 mmHg andor antihypertensive medications
Wamala *et al.* 2009^64^	2006	Uganda	Rukungiri	≥20	Both	Community-based	Systolic BP ≥ 140 mmHg and/or diastolic BP ≥ 90 mmHg andor antihypertensive medications
Grimsrud *et al*. 2009^65^	2002–04	South Africa	National survey	≥18	Both	Community-based	Self-report of hypertension diagnosis
Fezeu *et al*. 2008^66^	1994, 2003	Cameroon	Multi-centre	≥24	Both	Community-based	Systolic BP ≥ 140 mmHg and/or diastolic BP ≥ 90 mmHg andor antihypertensive medications
Maher *et al*. 2011^67^	2008–09	Uganda	NR	≥13	Rural	Community-based	Systolic BP ≥140 mmHg and/or diastolic BP ≥ 90 mmHg only
Ramirez *et al*. 2010^68^	2007	Malawi, Rwanda, Tanzania	Mwandama, Mayange, Mbola	≥18	Rural	Community-based	Systolic BP ≥ 140 mmHg and/or diastolic BP ≥ 90 mmHg or physician-diagnosed hypertension or antihypertensive medications
Adeke *et al*. 2022^69^	2017–18	Nigeria	Multi-centre	≥18	Both	Community-based	Systolic BP ≥ 140 mmHg and/or diastolic BP ≥ 90 mmHg or self-reported treatment using antihypertensive medications
Isezuo *et al*. 2011^70^	NR	Nigeria	Sokoto	15–65	Both	Community-based	Systolic BP ≥ 140 mmHg and/or diastolic BP ≥ 90 mmHg andor antihypertensive medications
Ejim *et al*. 2011^71^	NR	Nigeria	Imezi-Owa	40–70	Rural	Community-based	Systolic BP ≥ 140 mmHg and/or diastolic BP ≥ 90 mmHg andor antihypertensive medications
Solet *et al*. 2010^72^	2008	Mayotte	Mayotte	30–69	NR	Community-based	Systolic BP ≥ 140 mmHg and/or diastolic BP ≥ 90 mmHg andor antihypertensive medications
Ayodele *et al*. 2011^73^	2009	Nigeria	Osogbo	NR	Urban	Community-based	Systolic BP ≥ 140 mmHg and/or diastolic BP ≥ 90 mmHg or physician-diagnosed hypertension or antihypertensive medications
Hammami *et al*. 2011^74^	2008–09	Tunisia	Monastir	≥65	Both	Community-based	Systolic BP ≥ 140 mmHg and/or diastolic BP ≥ 90 mmHg or physician-diagnosed hypertension or antihypertensive medications
Njelekela *et al.* 2011^75^	NR	Tanzania	Mwanza	20–50	Urban	Community-based	Systolic BP ≥ 140 mmHg and/or diastolic BP ≥ 90 mmHg only
Ulasi *et al*. 2011^76^	2006	Nigeria	Enugu	NR	NR	Community-based	Systolic BP ≥ 140 mmHg and/or diastolic BP ≥ 90 mmHg only
Ogunniyi *et al*. 2011^77^	2001	Nigeria	Ibadan	≥70	Urban	Community-based	Systolic BP ≥ 140 mmHg and/or diastolic BP ≥ 90 mmHg only
Hendriks *et al.* 2012^78^	2009, 2010, 2011	Kenya, Namibia, Nigeria, Tanzania	Kwara, Nandi, Dar es Salaam, Greater Windoek	≥18	Both	Community-based	Systolic BP ≥ 140 mmHg and/or diastolic BP ≥ 90 mmHg andor antihypertensive medications
Musinguzi *et al*. 2015^79^	NR	Uganda	Mukono and Buikwe	≥15	Both	Community-based	Systolic BP ≥ 140 mmHg and/or diastolic BP ≥ 90 mmHg only
Menyanu *et al*. 2017^80^	2014–15, 2017–18	Ghana, South Africa	Multi-centre	18–50	Both	Community-based	Systolic BP ≥ 140 mmHg and/or diastolic BP ≥ 90 mmHg or physician-diagnosed hypertension
Akpa *et al.* 2020^11^	Multiple period	Burkina Faso, Ghana, Kenya, Mozambique, Namibia, Nigeria, South Africa, Sudan, Uganda & Zambia	Multi-centre	≥18	NR	Community-based	Systolic BP ≥ 140 mmHg and/or diastolic BP ≥ 90 mmHg or physician-diagnosed hypertension or antihypertensive medications
Mosha *et al*. 2017^81^	2013	Tanzania	National Survey	≥15	Both	Community-based	Systolic BP ≥ 140 mmHg and/or diastolic BP ≥ 90 mmHg only
Ezejimofor *et al.* 2016^82^	2014	Nigeria	Ebubu and Usokun	18–80	Rural	Community-based	Systolic BP ≥ 140 mmHg and/or diastolic BP ≥ 90 mmHg or antihypertensive medications
Mufunda *et al*. 2006^83^	2004	Eritrea	Multi-centre	15–64	Both	Community-based	Systolic BP ≥ 140 mmHg and/or diastolic BP ≥ 90 mmHg only
Gharbi *et al*. 2016^84^	2009–10	Morocco	El-Jadida and Khemisset	26–70	Both	Community-based	Systolic BP ≥ 140 mmHg and/or diastolic BP ≥ 90 mmHg only
Kamadjeu *et al*. 2015^85^	2015	Cameroon	National survey	≥15	Urban	Community-based	Systolic BP ≥ 140 mmHg and/or diastolic BP ≥ 90 mmHg or antihypertensive medications
Davids *et al*. 2019^86^	2008/09, 2014/16	South Africa	Bellville, Capetown	≥18	Urban	Community-based	Systolic BP ≥ 140 mmHg and/or diastolic BP ≥ 90 mmHg or antihypertensive medications
Bushara *et al*. 2016^87^	2015	Sudan	River Nile State	≥18	Urban	Community-based	Systolic BP ≥ 140 mmHg and/or diastolic BP ≥ 90 mmHg only
Okpechi *et al*. 2013^88^	2011–12	Nigeria	Abia	≥18	Both	Community-based	Systolic BP ≥ 140 mmHg and/or diastolic BP ≥ 90 mmHg or antihypertensive medications
Guwatudde *et al*. 2015^89^	2011–12	Uganda	Wakiso, Bushenyi	≥20	Both	Community-based	Systolic BP ≥ 140 mmHg and/or diastolic BP ≥ 90 mmHg or antihypertensive medications
Peer *et al*. 2013^90^	2008–09	South Africa	Cape Town	25–74	Urban	Community-based	Systolic BP ≥ 140 mmHg and/or diastolic BP ≥ 90 mmHg or antihypertensive medications
Sanuade *et al*. 2018^91^	2014	Ghana	Nationwide survey	15–49	Both	Community-based	Systolic BP ≥ 140 mmHg and/or diastolic BP ≥ 90 mmHg only
Oluyombo *et al*. 2014^92^	NR	Nigeria	Ekiti	≥18	Urban^b^	Community-based	Systolic BP ≥ 140 mmHg and/or diastolic BP ≥ 90 mmHg or antihypertensive medications
Soubeiga *et al*. 2017^93^	2013	Burkina Faso	National survey	25–64	Both	Community-based	Systolic BP ≥ 140 mmHg and/or diastolic BP ≥ 90 mmHg or antihypertensive medications
Msyamboza *et al*. 2012^94^	2009	Malawi	National survey	25–64	Both	Community-based	Systolic BP ≥ 140 mmHg and/or diastolic BP ≥ 90 mmHg or antihypertensive medications

BP, blood pressure; CAR, Central Africa Republic; NR, not reported.

^a^Hospital-based studies or datasets from hospital-based registries.

^b^Semi-urban.

### 3.2 Crude prevalence of hypertension in Africa

**Figure 3:**
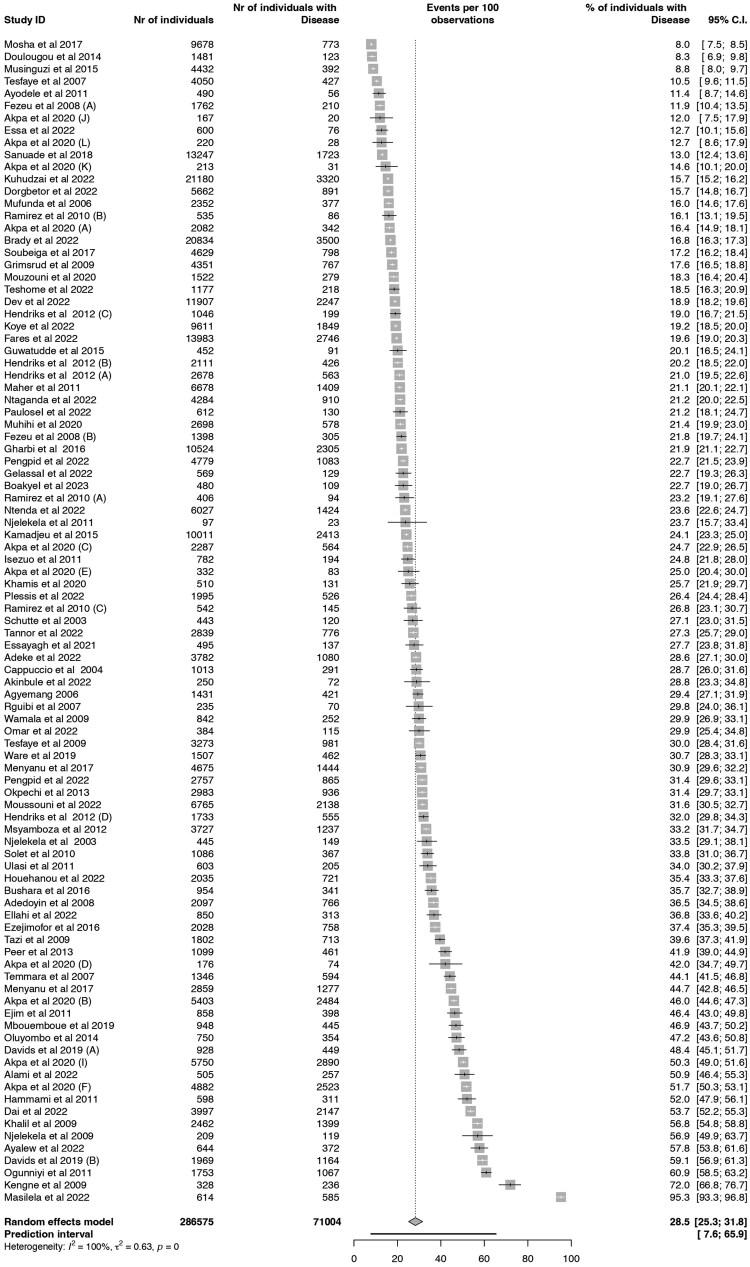
Forest plots for the pooled crude prevalence of hypertension per 100 persons in Africa.

The pooled crude prevalence of hypertension (per 100 persons) from the 78 studies published between 2003 and 2023 was 28.5% (95% CI: 25.3–31.8%), with very high heterogeneity (*I*^2^ = 100.0%) and 95% prediction intervals of 7.6–65.9 (*Figure 3*). The point estimate of crude hypertension prevalence for studies recruiting respondents from urban settings only was higher, 32.9% (95% CI: 26.8–39.5%, *I*^2^ = 99.0%) compared with studies from rural areas only; 26.3% (95% CI: 20.4–33.3%, *I*^2^ = 99.0%) or both; 26.5% (95% CI: 21.5–31.2%, *I*^2^ = 100.0%) (see Supplementary material online, *Figure S1*).

For studies reporting data by sex, the pooled prevalence was 27.9% (95% CI: 23.9–32.2%, *I*^2^ = 98.9%) in males and 28.1% (95% CI: 23.9–32.7%, *I*^2^ = 99.3%) in females with a within-study risk difference (see Supplementary material online, *Figure S2*) of −0.4% (95% CI: −2.0 to 1.2%). The risk difference was slightly higher among studies from urban settings: −2.4 (95% CI: −5.8 to 1.0%) than studies from rural areas: 0.0% (95% CI: −1.9 to 1.9%). In subgroup analysis by age (see Supplementary material online, *Figure S3*), a significant (*P* < 0.001) difference was observed with an increasing prevalence across age groups. The pooled prevalence was 9.1% (95% CI: 6.9–12.0%) in people aged <35 years and increased to 51.4% (95% CI: 42.0–60.6%) in people aged 75 years or older.

The analysis was stratified accordingly to test the robustness of the pooled estimates to address the potential differences in hypertension reporting across study settings (hospital-based/community-based). Generally, *I*^2^ was >99.0%, and the point estimate for the crude prevalence of hypertension was 27.2% (95% CI: 24.5–30.1%) for community-based studies, but 56.0% (95% CI: 23.0–84.4%) for hospital-based studies. Due to the observed variations in the diagnostic criteria for hypertension, additional analysis was conducted by first excluding studies where hypertension diagnosis was based on self-report only. The crude prevalence of hypertension was 27.3% (95% CI: 23.9–31.0%) for studies with hypertension definitions, including systolic BP ≥ 140 mmHg and/or diastolic BP ≥ 90 mmHg and/or antihypertensive medications, 29.5% (95% CI: 21.9–38.5%) for systolic BP ≥ 140 mmHg and/or diastolic BP ≥ 90 mmHg or physician-diagnosed hypertension or antihypertensive medications, and 29.2% (95% CI: 20.6–39.5%) for studies with systolic BP ≥ 140 mmHg and/or diastolic BP ≥ 90 mmHg only. A sensitivity analysis (although it is of limited utility in the light of the high heterogeneity and large number of studies included) was carried out, and the pooled crude prevalence estimates ranged from 26.8 to 27.5%, and the *I*^2^ values remain very high (99.5%).

**Figure 4:**
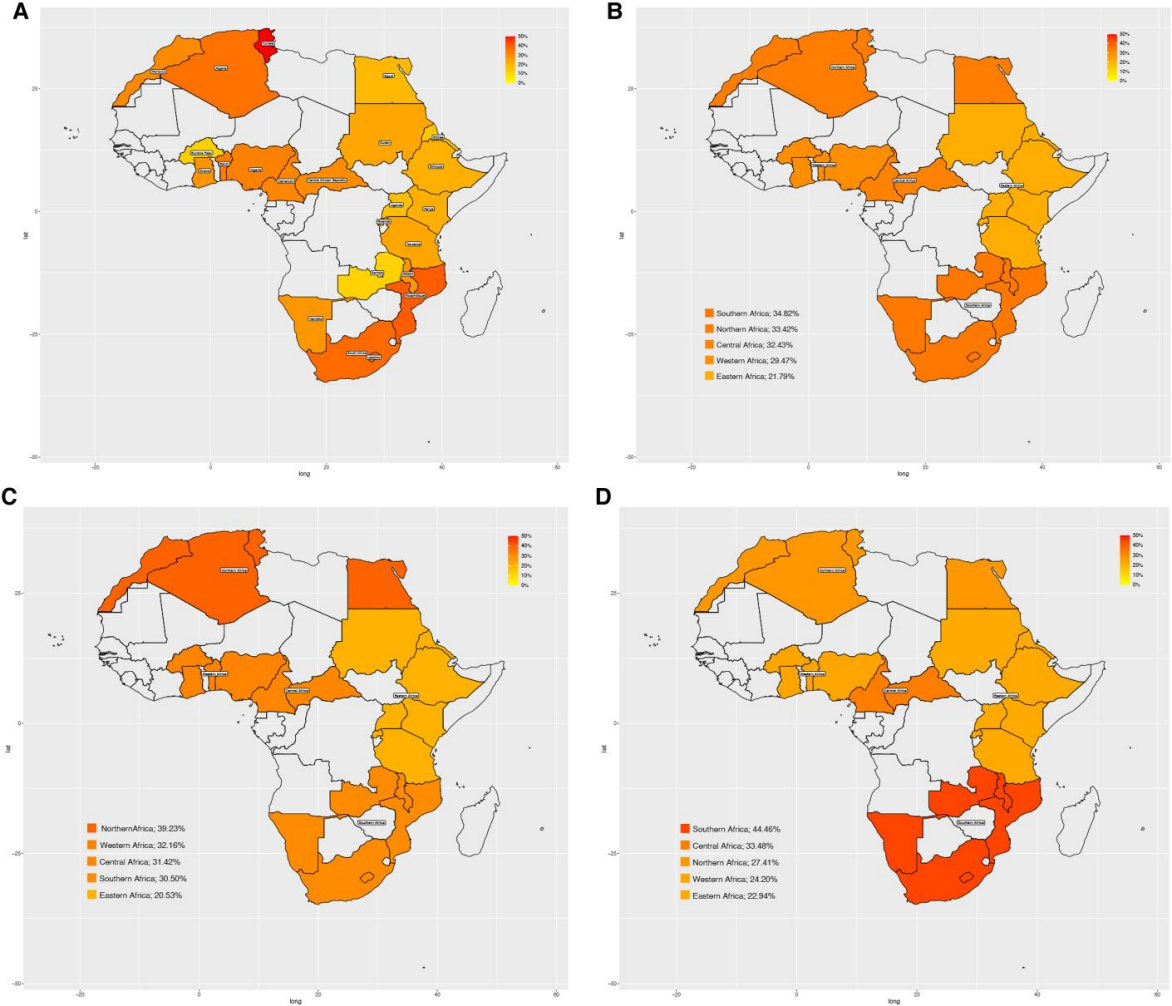
Distribution of the crude prevalence of hypertension by country-specific estimates in the overall study (*A*), by region of Africa (*B*), between 2002 and 2012 (*C*) and after 2012 (*D*).

Overall, the country-specific distribution of hypertension varied widely (*Figure 4A*). However, the regional patterns of hypertension burden in Africa (*Figure 4B*) suggest a 34.8% prevalence in Southern Africa and a 21.8% crude prevalence in East Africa. Given the meta-analysis focused on studies published in the last two decades (2002–23), crude prevalence varied widely after stratifying by 10-year intervals of study participant recruitment (before or after 2012). Specifically, the point estimate of the prevalence of hypertension was highest in the North (39.2%) and West (32.2%) African regions but lowest in the East African region (20.5%) among studies conducted between 2002 and 2012 (*Figure 4C*). However, studies conducted post-2012 (*Figure 4D*) suggest the point estimate of hypertension prevalence was highest in Southern (44.5%) and Central (33.5%) Africa but remained generally lowest in Eastern Africa (22.9%). The overall burden did not change significantly between the two decades: 28.9% before 2012 and 27.7% after 2021.

### 3.3 Age-adjusted prevalence of hypertension in Africa

**Figure 5:**
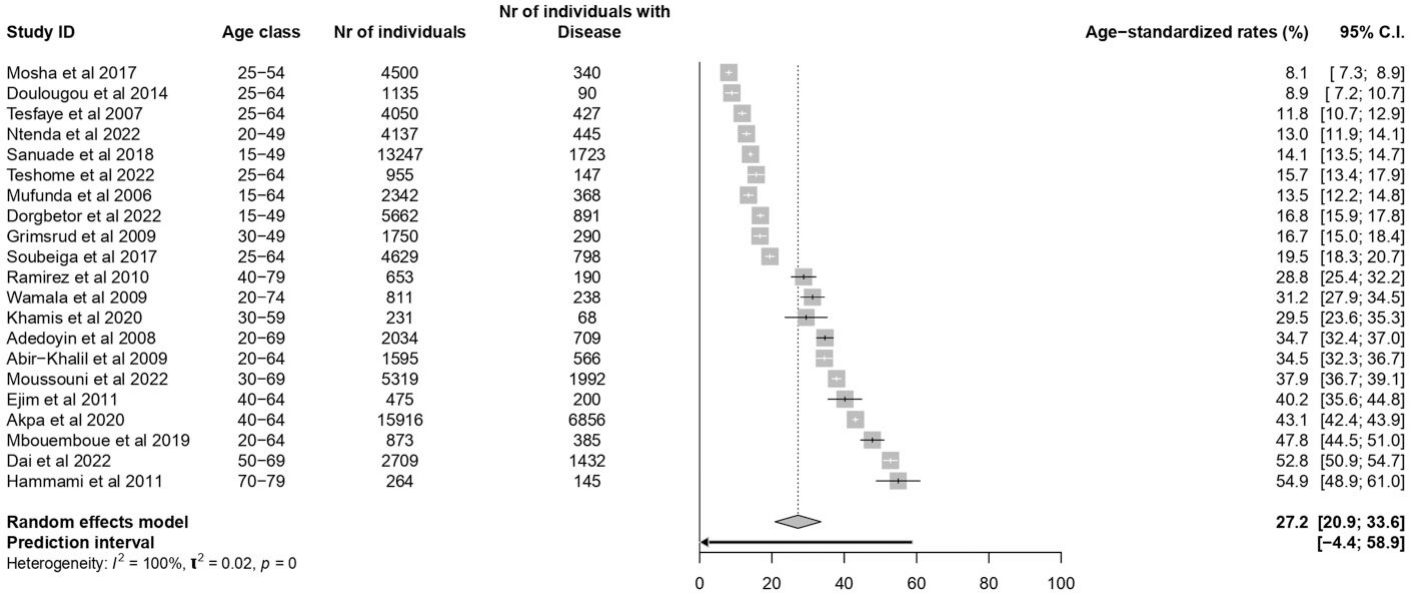
Age-adjusted prevalence of hypertension in Africa.

In a subset of 21 articles reporting age stratification consistent with the WHO standard population (*Figure 5*), the pooled age-standardized prevalence was 27.2% (95% CI: 20.9–33.6%) in Africa.

### 3.4 Publication bias

Supplementary material online, *Figure S4* shows the funnel plot for the overall meta-analysis. The visual inspection of the plot did not show any substantial violation of symmetry (except for a study from South Africa^43^); the Egger test confirmed the absence of publication bias (bias = 5.2, *P* = 0.09). However, most individual study estimates were outside the 95% pseudo-confidence limits, thus providing further evidence of the significant heterogeneity observed.

## 4. Discussion

This systematic review pooled epidemiological data on the crude prevalence of hypertension from cross-sectional studies across Africa, including 71 004 hypertension cases from a total population of 286 575 conducted in 23 countries (out of a total of 54 African countries), leaving most of the world's second most populous continent short of high-quality data critical for evidence-based decision-making to prevent, track, and manage hypertension.

Approximately one in every three Africans has hypertension, with prevalence varying across African regions. Africa is the epicentre of global hypertension, and its prevalence is expected to be increasing.^95^ Worldwide, the number of people between 30 and 79 years old with hypertension doubled, and there are substantial variations in age-adjusted prevalence across countries and world regions, with abysmal treatment and control rates in most countries in sub-Saharan Africa.^96^ Also, our findings were relatively comparable with that among adults 18 years and above in the USA, where the age-adjusted prevalence of hypertension decreased within two decades, from 47.0% in 1999–2018 to 45.4% in 2017–18.^97^ The case is not different in Central and Eastern Europe, where the age-standardized prevalence of hypertension remains >30% among women and >40% among men.^96^ Among Canadian adults, the prevalence of hypertension (defined as drug treatment for high BP or BP ≥ 140/90 mm Hg) was proportionately lower (22.6%) and better controlled (68.1%)^98^ than in most African countries, as observed in our study where the crude prevalence was 28.5% with 7% controlled.^99,100^ Hypertension prevalence among older adults in Africa is still very high, as found in some systematic reviews and meta-analyses a few years ago.^101,102^ This is primarily due to the persistently low detection and treatment rates of hypertension in Africa.^96^ Globally, there are inequalities in hypertension prevention and control,^103^ but the common trend is a higher hypertension prevalence in LMIC compared with high-income countries.^104^ A recent report has also shown that the WHO African Region has the highest prevalence of hypertension, while the WHO Region of the Americas has the lowest prevalence of hypertension.^15^ The lopsided burden in Africa has been attributed to increasing population growth, ageing, rising urbanization, unhealthy lifestyles such as malnutrition, and low physical activity.^101^ This same pattern has also been reflected in the intracontinental prevalence disparity between the rural and the urban regions^95^ and age differences as in this study. Contributing to the poor control is the lack of a sustainable, well-coordinated, integrated approach that would involve all stakeholders at the various stages and levels of hypertension surveillance, screening, and treatment.^105^ In addition, there is little to no awareness of access and adherence to a unified socioeconomically and culturally implementable hypertension guideline in Africa.^106^

### 4.1 Gaps in existing data

The absolute estimates of the hypertension burden in Africa are still unknown, and contrary to the projected increase in hypertension prevalence,^107^ our study revealed a modest estimate and trend from the cross-sectional studies we reviewed. This could be because the epidemiological studies selected did not cut across all those countries or regions where lifestyles, among other cardiovascular risks, have been almost entirely Westernized.^108^ Also, a significant proportion of the retrieved studies were excluded, as shown in the PRISMA flow chart, due to unclear or poorly reported methodologies, and in some cases, the findings were incoherent with the statistics in the reported dataset. Also, in the selected studies, we observed high levels of heterogeneity attributable to the varying methodologies (including sampling strategy, inclusion criteria, and criteria for hypertension definition, among others) and populations studied, as with other similar meta-analyses of prevalence studies,^109,110^ where high heterogeneities have been noted. These shortcomings allude to multifactorial constraints from limited skills, human resources shortage, and inadequate access to infrastructure and resources to execute robust and high-ended epidemiological studies. As a result, there is an increased tendency to conduct hospital-based studies. Although applicable to some extent, hospital studies lack applicability to the population as a whole compared with community-based reports.^111^

### 4.2 Strengths, limitations, and future directions

Although more than 98% of the studies reviewed reported the diagnostic criteria, including BP measurement, inconsistencies remain in ensuring precision to minimize systematic errors in hypertension diagnosis. Even though variations in diagnosing hypertension did not significantly alter the overall pooled estimates in this study, it is imperative to clarify the significance of consistency in BP measurements and hypertension diagnosis in future studies to track hypertension accurately. In addition, most studies did not pay attention to undiagnosed hypertension despite it being a significant public health challenge, especially in LMIC. This would be valuable for future studies to assess the profile, magnitude, and risk factors of undiagnosed hypertension in sub-Saharan Africa. Given the limited data, this current study could not discern the temporal trends in hypertension prevalence for the years under review, which future studies should consider to offer valuable insights into the broader epidemiological patterns of hypertension.

The substantial hypertension burden observed in this study underscores the need for stakeholders to prioritize an agenda for Africa-wide epidemiological and surveillance initiatives for effective hypertension management. Furthermore, geographic, anthropometric, lifestyle, genetic, and epigenetic influences on hypertension,^112^ could not be fully assessed^113^ due to the underrepresentation of various regions and cultures. Therefore, standardized guidelines/protocols for CVD epidemiological studies, particularly those addressing hypertension, should be developed across Africa.

Translational perspective Hypertension is the most common modifiable risk factor for cardiovascular diseases, with increasing attributable morbidity and mortality, but the actual burden of hypertension is poorly characterized in Africa. To our knowledge, this study provides the most comprehensive evaluation of epidemiological studies on hypertension in Africa from the last two decades. By systematically reviewing and pooling data, we presented Africa-wide and regional estimates of hypertension prevalence, offering crucial information to guide the efficient allocation of limited resources for hypertension prevention and control across the continent. Our study expands on previous efforts to characterize hypertension burden by providing recent trends by age group and urban–rural and regional differences in Africa. This study demonstrates that the burden of hypertension in Africa remains substantial and is among the highest in the world, with limited progress in achieving meaningful reductions. Concerning trends are evident, affecting populations across various age groups and residential settings. These estimates may assist policymakers in strategizing a path towards achieving health and well-being for all in keeping with the Sustainable Development Goal 3 and Africa 2063 agenda, the master plan for positioning Africa into the global powerhouse in the future.

## 5. Conclusion

The burden of hypertension remains high in Africa, especially among older adults. Evidence from our study's screening process, following PRISMA guidelines, highlighted inadequacies in current epidemiological study protocols. The outcomes from these studies, used for planning previous hypertension control programmes, may have contributed to significant implementation failures. This may explain why hypertension prevalence continues to rise despite ongoing interventions. Therefore, rigorous, robust, collaborative multi-country-funded studies are necessary to generate evidence, utilizing existing government census data across various African countries to accurately assess the current burden of hypertension.

## Supplementary Material

cvaf125_Supplementary_Data

## Data Availability

The search strategy, included studies, data extracted, analysis plans, quality assessment, assessment of the publication bias, and relevant data are available in the article. Also, the protocol is available in PROSPERO.
